# Single-stage bilateral thoracic surgery via a combined VATS and open approach for left central bronchogenic carcinoma with carinal invasion: report of two cases

**DOI:** 10.1186/s13019-015-0276-z

**Published:** 2015-05-21

**Authors:** Bo Ai, Yongde Liao, Zheng Zhang, Xiangning Fu

**Affiliations:** Department of Thoracic Surgery, TongJi Hospital, TongJi Medical College, Huazhong University of Science and Technology, 1095 Jiefang Ave, Wuhan, 430030 China

**Keywords:** Left central lung cancer, Carinal resection, Single-stage bilateral surgery, VATS

## Abstract

**Background:**

Surgery for patients with left central bronchogenic carcinoma invading the carina is challenging due to the complexity of left sleeve pneumonectomy, carinal resection, and airway reconstruction and management. Here we describe a modified approach to overcome this problem.

**Case presentation:**

Between March 2011 and September 2012, two patients with left central bronchogenic carcinoma invading the carina underwent single-stage bilateral thoracic surgery via a combined approach incorporating video-assisted thoracic surgery (VATS) and thoracotomy in our hospital. We reviewed our experience with this type of surgery and analyze its outcomes.

**Conclusions:**

Single-stage, bilateral thoracic surgery incorporating video assisted thoracic surgery (VATS) and thoracotomy provides optimal exposure of the operative field, reduces surgical trauma, and ensures the integrity of tumor excision and exactness of tracheobronchial anastomosis. This may be a safe and feasible alternative for left carinal pneumonectomy.

## Background

The treatment of left central bronchogenic carcinoma invading the carina is challenging due to the complexity of left sleeve pneumonectomy, carinal resection and airway reconstruction and management [1, 2]. The main approaches used for this surgery include left posterolateral thoracotomy and bilateral thoracotomy. However, the former provides poor exposure, while the latter causes massive surgical trauma and is associated with severe postoperative complications and high mortality rates [3]. Thus, it is necessary to develop a surgical approach that provides optimal exposure and reduces surgical trauma. In March 2011 and September 2012, two of those patients underwent sequential right thoracotomy and left video assisted thoracic pneumonectomy in the Department of Thoracic Surgery, Tongji Hospital, Hubei, China. In this report, we review our experience with this type of surgery and analyze its outcomes.

## Case presentation

### The pre-operative data

**Fig. 1:**
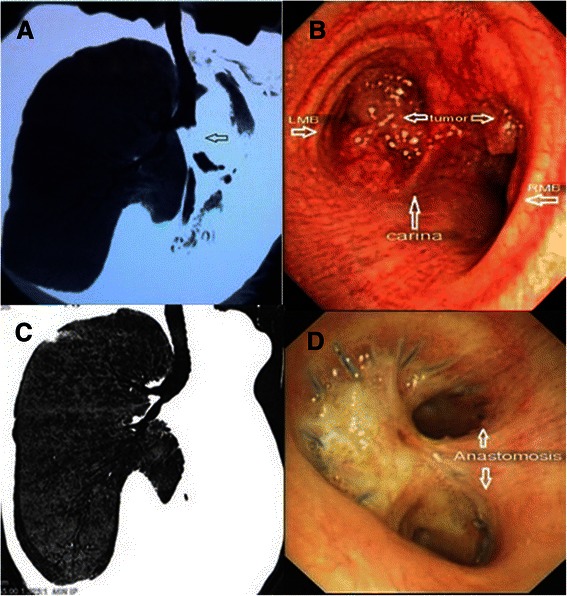
**a** The tumor in the left main bronchus extended up to the bifurcation of the upper and lower lobe orifices. Atelectasis of the left lung was seen. **b** Tumor involved the carina and proximal part of the right main bronchus. **c** CT showed an unobstructed airway at 19 months after the operation. **d** Bronchoscopy reveald an intact anastomosis, smooth mucosa

The first case was a 50-year-old man with a history of cough and hemoptysis for 6 months and dyspnea for two months was referred to our hospital. Chest computed tomography (CT) and bronchoscopy showed a mass in the left main bronchus with atelectasis of the left lung. The mass extended up to the bifurcation of the upper and lower lobe orifices, and involved the carina and proximal portion of the right main bronchus (Fig. 1a and b). Histological examination of a biopsy specimen confirmed the diagnosis of pulmonary carcinoma. Preoperative lung-function tests demonstrated that the forced expiratory volume in 1 s (FEV1) was 1.93 L, the ratio of FEV1 to forced vital capacity (FEV1/FVC) was 59 %, and the maximum ventilatory volume (MVV) was 63 L/min. No distant metastases were found on fluorodeoxyglucose-positron emission tomography/CT scans.

The other case was a 59-year-old man with a history of dry cough for more than four months was admitted to our department. Chest CT showed a mass that was located in the lower lobe of the left lung and involved the left main bronchus and carina. Bronchoscopy revealed a neoplasm occluding the left lower lobe bronchus and involving the left main bronchus, carina, and proximal portion of the right main bronchus. Histological examination revealed a squamous cell carcinoma. Pulmonary function tests demonstrated that FEV1, FEV1/FVC, and MVV were 3.4 L, 67 %, and 83 L/min, respectively. No distant metastases were observed.

### Operative technique

Under combined general intravenous and inhalation anesthesia, independent lung ventilation was achieved through a double-lumen tube, and during airway reconstruction, patients were intermittently ventilated through cross-field right bronchial intubation.

**Fig. 2:**
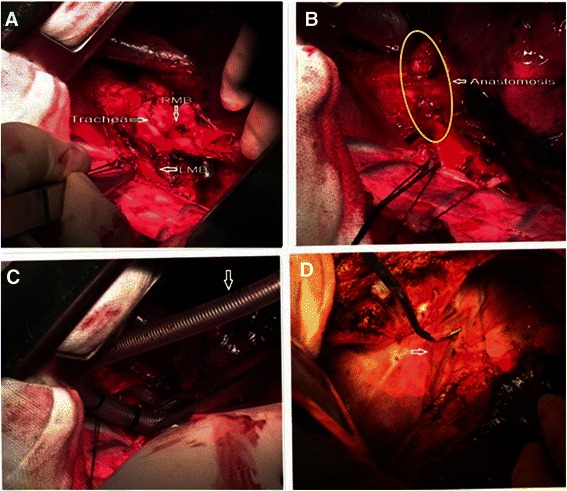
**a** The lower portion of the trachea, the carina, the left main bronchus, and the right main bronchus were exposed via a right thoracotomy. **b** The trachea and right main bronchus were anastomosed in an end-to-end fashion. **c** Ventilation through right bronchial intubation across the operative field. **d** pedicled azygos vein flap which were to completely encircle the anastomotic stoma

Both patients underwent single-stage, bilateral thoracic surgery via a combined approach incorporating thoracotomy and VATS. After left lateral decubitus positioning, surgery was performed through right posterolateral thoracotomy in the fifth intercostal space including carinal resection, airway reconstruction, and systemic lymph node dissection. The trachea was resected approximately 1 cm above the carina, and the right main bronchus was transected at its distal portion. Both surgical margins were found to be cancer free on intraoperative frozen-section biopsy. The trachea and right main bronchus were anastomosed in an end-to-end fashion (Fig. 2a and b) using continuous 3–0 surgipro sutures (Covidien, Surgipro II).

During the anastomosis, the patients were ventilated intermittently through right main bronchus intubation across the surgical field (Fig. 2c): the ventilation tube was inserted into the distal end of the divided right main bronchus for SPO_2_ to reach 100 %, so it can be withdrawn and the anastomosis can be performed starting from the posterior wall of the trachea. Once the SPO_2_ drops below 90 %, the surgeon should stop and reinsert the tube to elevate the SPO_2_ again and continue the anastomosis. Roughly, it takes 3 cycles to complete the anastomosis. When the anastomosis was completed, the surgical-field endotracheal intubation was replaced with orotracheal intubation, with the endotracheal tube passing through the anastomosis site and into the right main bronchus.

Azygos vien was transected after ligation. While its distal end was ligated, the other end was incised longitudinally to be used as a pedicle flap (Fig. 2d) to encircle the site of anastomosis. Freeing the left main bronchus from the surrounding was additionally done to prepare for VATS left pneumonectomy.

**Fig. 3:**
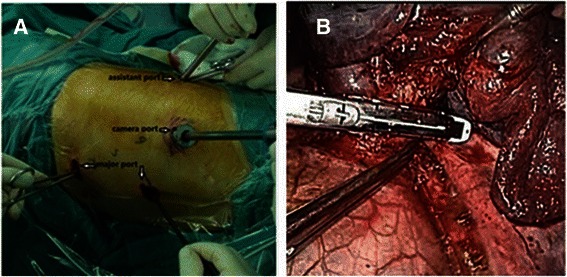
**a** Four thoracic ports were used for left thoracoscopy. **b** VATS left sleeve pneumonectomy: the left pulmonary artery was ligated and divided with an endoscopic stapler

When the operation on the right side was completed, VATS and left sleeve pneumonectomy were performed with the patients in the right lateral position. Four thoracic ports were used for left thoracoscopy: The camera port was located at the 8th intercostal space in posterior axillary line, the major operating port between the 4th and 6th intercostal space in anterior axillary line, and an assistant port at the 7th intercostal space in scapular line (Fig. 3a). Since the left main bronchus had been dissociated freely and the subcarinal lymph nodes had been dissected during the right thoracotomy, the left sleeve pneumonectomy was performed after dividing the left pulmonary artery and vein with linear staplers. In addition, the left hilar lymph nodes and the lymph nodes at stations 5–9 were dissected (Fig. 3b).

The total operating hours of two cases were 5.5 h and 6 h separately. In the first case, the right thoracotomy took 3.5 h and VATS left pneumonectomy took 2 h, while the first stage of the other case lasted for 4 h and the second one lasted for 2 h.

### The post-operative outcome

Surgical outcomes and follow-up: For the first patient, postoperative histopathological examination of the surgical specimens revealed an adenoid cystic carcinoma in the left main bronchus, tumor-free margins, and metastasis-free lymph nodes at each station examined. The pathological stage was pT4N0M0, IIIA (7th UICC guidelines, 2009). Histopathological examination in the second patient revealed a moderately-to-poorly differentiated squamous cell carcinoma in the left lung with metastasis to the lymph nodes on the left side at station 8 (1/1), station 10 (1/8), and station 7 (1/1). The other lymph nodes and the section margins were negative for cancer cells. The pathological stage was pT4N2M0, IIIB (7th UICC guidelines, 2009).

The first patient was weaned off postoperative ventilator-assisted breathing after 6 h, and his respiratory functions were well recovered. The patient was ambulated on postoperative day (POD) 2 and discharged on POD 12. A follow-up examination at 19 months showed no recurrence, metastasis, chest pain, or dyspnea. The patient could engage in light physical work and had a good quality of life. Both the postoperative CT scan and bronchoscopy revealed an unobstructed airway (Fig. 1c and d). The second patient was weaned off the ventilator after 5 h, and showed a recovery of the pulmonary functions, but he developed right lung infection and underwent tracheostomy on POD 7; beside Bronchofiberscopy revealed an integrated non leaking anastomosis. Unfortunately, after initial recovery of the lung infection, he suffered from a sudden respiratory tract bleeding and suffocation and died on POD 20. An autopsy was not performed. The clinical cause of death was considered to be a blood vessel rupture after erosion by local infection.

## Discussion

Left central lung cancer involving the carina or even the right main bronchus poses many challenges during thoracic surgery, as it requires special surgical techniques and airway management, including left sleeve pneumonectomy, carinal resection and airway reconstruction. Carinal resection and airway reconstruction via a left thoracotomy are extremely difficult, limited exposure due to aortic arch covering the left main bronchus and deep operating site due to natural right deviation of the carnia. Therefore, right thoracotomy is commonly added to left sleeve pneumonectomy [4, 5], but such one stage bilateral thoracotomy is associated with severe surgical trauma, loss of pulmonary function, postoperative respiratory failure, and high mortality rates [3]. Bilateral thoracotomy through a transverse sternotomy via a clamshell incision has been reported as an alternative surgical approach for carinal and airway reconstruction surgery [6]. However, this approach has some disadvantages, such as severe surgical trauma and the lack of upper mediastinal lymph node dissection. Thus, left central lung cancer involving the carina or right main bronchus has been considered a forbidden zone in thoracic surgery.

This study showed that a right thoracotomy followed by left video assisted pneumonectomy might be a safe and feasible alternative method. Although the second patient who suffered sudden respiratory tract bleeding and suffocation died on POD 20, his lung function recovered well postoperatively. Similar to sequential posterolateral thoracotomies, such approach offers both optimal space and exposure for proper airway resection and reconstruction as well as radicalness of resection for the tumor and the lymph nodes. Additionally, comparing our surgery to previous trial of performing VATS carinal pneumonectomy, it makes the surgery less invasive producing less trauma by smaller size of incision and resolves the ventilation problem during air way reconstruction by special intermittent ventilation through right main bronchus intubation across the operation field.

This combined approach incorporating VATS and thoracotomy extends the applications of conventional thoracic surgery. Candidates to left carinal pneumonectomy who have left central bronchogenic carcinoma invading carina and right main bronchus should have no invasion of distal right main bronchus or distant metastasis and should have sufficient cardiac and pulmonary reserve.

The key points of this surgical procedure are as follows: (1) During the right thoracotomy, the tumor and left main bronchus should be dissociated sufficiently to facilitate surgical manipulation during VATS and left sleeve pneumonectomy. (2) The lower section of the trachea and right main bronchus must be resected about 1 cm away from the carina, and en bloc tumor resection must be achieved during the left-sided surgery. Intraoperative frozen-section pathological examination should be used to confirm negative resection margins for the trachea and right main bronchus. (3) The surgeon and anesthetist should cooperate well during the anastomosis, the patients are ventilated intermittently through right main bronchus intubation across the surgical field. (4) The tracheobronchial anastomosis should be circularly embedded within a pedicled azygos vein flap to prevent bronchopleural fistula. (5) The vagus nerves should be protected during both the right- and left-sided surgery, as these nerves are required to restore gastrointestinal function postoperatively.

## Conclusions

Single-stage, bilateral thoracic surgery via a combined approach incorporating VATS and thoracotomy is a modified approach that maintains the advantages of bilateral thoracotomy yet avoids the marked surgical trauma associated with rib spreading during left posterolaterial thoracotomy. Therefore, this surgical approach might be a safe and feasible alternative for the management of left central bronchogenic carcinoma involving the carina or right main bronchus. However, our findings should be confirmed in a greater number of patients.

## Consent

Written informed consent was obtained from the patient for the publication of this report and any accompanying images.

## Abbreviations

VATS: Video assisted thoracic surgery
CT: Computed tomography
FEV1: Forced expiratory volume in 1 s
FVC: Forced vital capacity
MVV: Maximum ventilatory volume
POD: Postoperative day
